# Endogenous DAF-16 spatiotemporal activity quantitatively predicts lifespan extension induced by dietary restriction

**DOI:** 10.1038/s42003-023-04562-2

**Published:** 2023-02-20

**Authors:** Javier Huayta, Joseph P. Crapster, Adriana San-Miguel

**Affiliations:** Department of Chemical and Biomolecular Engineering, North Carolina State University, Raleigh, NC USA

**Keywords:** Ageing, Image processing, Genetic engineering

## Abstract

In many organisms, dietary restriction (DR) leads to lifespan extension through the activation of cell protection and pro-longevity gene expression programs. In the nematode *C. elegans*, the DAF-16 transcription factor is a key aging regulator that governs the Insulin/IGF-1 signaling pathway and undergoes translocation from the cytoplasm to the nucleus of cells when animals are exposed to food limitation. However, how large is the influence of DR on DAF-16 activity, and its subsequent impact on lifespan has not been quantitatively determined. In this work, we assess the endogenous activity of DAF-16 under various DR regimes by coupling CRISPR/Cas9-enabled fluorescent tagging of DAF-16 with quantitative image analysis and machine learning. Our results indicate that DR regimes induce strong endogenous DAF-16 activity, although DAF-16 is less responsive in aged individuals. DAF-16 activity is in turn a robust predictor of mean lifespan in *C. elegans*, accounting for 78% of its variability under DR. Analysis of tissue-specific expression aided by a machine learning tissue classifier reveals that, under DR, the largest contribution to DAF-16 nuclear intensity originates from the intestine and neurons. DR also drives DAF-16 activity in unexpected locations such as the germline and intestinal nucleoli.

## Abstract (web summary)

The spatiotemporal activity of the DAF-16 transcription factor correlates with *C. elegans* population lifespan after dietary restriction exposure.

## Introduction

*C. elegans* exposed to plentiful food and a stress-free environment can sustain growth and reproduction. Under harsh environmental conditions such as food deprivation, oxidative and heat stress, and others, several genetic pathways activate to promote cell protection and survival in *C. elegans*. These pathways are governed by transcription factors such as DAF-16, PHA-4, and SKN-1^1^. Previous work has shown that these genetic pathways can be manipulated to modulate gene expression and extend lifespan^2–4^. Dietary restriction (DR) is the best-known environmental factor that can extend lifespan in many species^5–8^. In *C. elegans*, DAF-16 is a key pro-longevity transcription factor that regulates the Insulin/IGF-1 signaling pathway. Under various forms of stress, DAF-16 migrates from the cytoplasm to the nucleus where it can regulate gene expression programs that lead to lifespan extension^9,10^. DAF-16 has been shown to be activated by DR, but only under certain regimes^11–13^. Likewise, systems approaches that have been used to identify and manipulate the regulatory networks of the DR response, confirm the involvement of DAF-16^14^. However, how large is the influence of DR on DAF-16 activation and its subsequent effect on lifespan extension has not been quantitatively determined. In addition, whether the spatiotemporal activity of key aging transcription factors is sufficient to predict the lifespan extension conveyed by their stress-induced activities is still unclear. Elucidating the contributions of these components to *C. elegans* survival would permit interventions that target lifespan augmentation and shed light on the relative importance of different pathways towards longevity. With these considerations, we analyze lifespan in *C. elegans* as the outcome of cumulative molecular activity of DAF-16 prompted by exposure to DR.

Various approaches have been successfully used to probe gene expression in *C. elegans’* cells, tissues, and at different developmental stages, such as DNA microarrays, real-time PCR, RNAseq, and serial analysis of gene expression (SAGE)^15–19^. Since these methods are destructive in nature, as they require extraction of DNA and RNA, they are not suitable to obtain the spatial information required to assess nuclear translocation of cytoplasmic DAF-16. Previous studies have also used strains with fluorescently tagged DAF-16, which carry transgenic arrays that typically have hundreds of copies of the gene^20^ and label only specific isoforms^21–23^. Here, we measure endogenous activity of all DAF-16 isoforms by tagging the *daf-16* locus at the 3' end using CRISPR/Cas9^24^. Coupled with spatiotemporal quantification of nuclear fluorescence through quantitative image analysis and machine learning, this approach enables in vivo analysis of all spatiotemporal DAF-16 abundance in *C. elegans* at endogenous levels, in a tissue-specific manner. This approach reveals that DAF-16 spatiotemporal activity (measured as lifelong total nuclear intensity) is a strong predictor of lifespan in *C. elegans* populations exposed to DR in liquid culture, accounting for 78% of DR-induced lifespan variability. Furthermore, we show that the main contributors to this DAF-16 activity are neurons and intestinal cells, indicating that the observed lifespan extension originates mainly from the translocation and activity of DAF-16 in these cell types. Finally, we demonstrate that DAF-16 activity is observed in unexpected locations such as germ cells and intestinal nucleoli, where DAF-16 could be undergoing yet to be described interactions affecting the aging process.

## Results

### DR regimes modulate endogenous DAF-16 spatiotemporal abundance

**Fig. 1:**
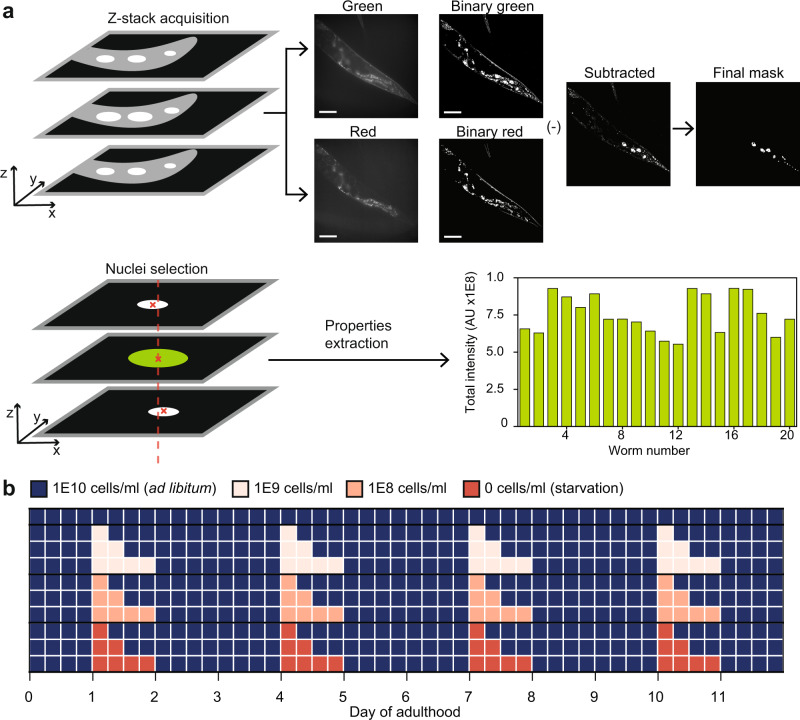
Quantitative analysis of endogenous DAF-16 under dietary restriction regimes. **a** Four Z-stacks of 30 slices are taken of each animal. Each slice in the green channel has a corresponding red channel slice. Both are converted to binary images and subtracted from each other to eliminate autofluorescence. The resulting image is treated with morphological functions and objects are filtered based on extent, solidity, and eccentricity. An image containing only cell nuclei is obtained, and largest nuclei are retained if they appear in several slices. Pixel-based intensity is then aggregated to obtain total DAF-16 intensity in all of the animal’s identified nuclei. **b** Diagram of DR regimes tested in this study. Animals were exposed to three levels of DR: 1E9, 1E8, or 0 OP50 cells/ml; for three exposure times: 6, 12, or 24 h. After each exposure, animals were put back in an *ad libitum* concentration (1E10 OP50 cells/ml). Exposure to DR was performed at Days 1, 4, 7, and 10 of adulthood. Scale bars are 50 µM.

To study the quantitative link between endogenous DAF-16 nuclear abundance, and lifespan, we first aimed to analyze the response of endogenous DAF-16 to multiple DR regimes by varying food concentration and exposure time. Using CRISPR/Cas9, we generated a strain with the endogenous *daf-16* locus labeled with GFP at the 3' end, and measured the response of DAF-16 using a custom image processing algorithm that quantifies nuclear intensity throughout the entire animal. Accurate analysis of DAF-16::GFP levels is challenging, as single copy reporters lead to extremely dim images. Since GFP intensities from DAF-16 were lower or similar to those from autofluorescent lipid droplets, we performed image acquisition in the green and red channels (Fig. 1a), allowing subtraction of autofluorescence from the GFP signal. Analysis was performed on every slice of a z-stack covering the entire animal, and four separate z-stacks per channel were acquired per animal to cover the entire worm length. Information from the four z-stacks were aggregated for each worm.

**Fig. 2:**
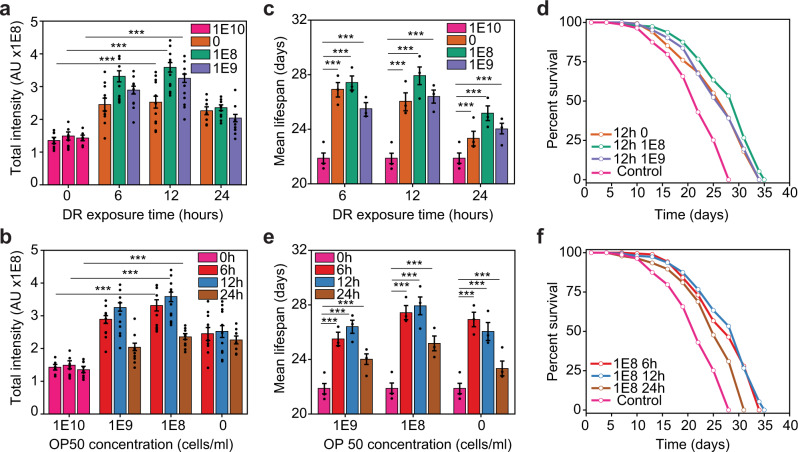
DR regimes modulate endogenous DAF-16 activity. **a** DAF-16 total intensity as a function of exposure time to DR for various food concentrations at Day 1 of adulthood. **b** DAF-16 total intensity as a function of food concentration for various exposure times at day 1 of adulthood. **c** Mean lifespan as a function of exposure time at various food concentrations. **d** Lifespan curves for populations under various food concentrations with 12 h of exposure to DR. **e** Mean lifespan as a function of food concentration at various exposure times. **f** Lifespan curves for populations under various exposure times to DR with a 10^8^ OP50 cells/ml food concentration. *p* < 0.001 (***). Error bars are SEM. All *p*-values were calculated using Tukey HSD for all pairwise comparisons after one-way ANOVA (unequal variances) comparison. Control population (pink) is *ad libitum* (1E10 cells/ml, 0 h exposure time).

Animals were exposed to DR conditions at Day 1 of adulthood and every third day thereafter (Fig. 1b). All DR conditions induced an increase in the total nuclear intensity of DAF-16 compared to well-fed animals used as a control (Fig. 2a, b). The strongest DAF-16 response, as measured by DAF-16::GFP nuclear localization, was observed for intermediate DR exposure times, peaking at 12 h. The longest exposure (24 h) showed a higher response than the control, but not as pronounced as 6 and 12 h. Noticeably, we observed a subtle DAF-16 nuclear localization on well-fed animals. A previous study demonstrated that DAF-16 is activated during the normal aging process but starting at Day 4 of adulthood^25^. However, we observed that transferring animals from solid media to liquid culture induces DAF-16 nuclear localization even when well-fed (Fig. S3). We theorize that this is an effect of fatigue-related stress produced by swimming^26^. To test that the addition of the GFP tag to DAF-16 does not disrupt its function, we analyzed expression of target genes and lifespan in a *daf-2* mutant background, where DAF-16 is constitutively active^27,28^. As expected, *sod-3* and *mtl-1*, which DAF-16 induces^29^, are upregulated, while *dod-17*, known to be inhibited by DAF-16^30^, is downregulated (Fig. S4a). Likewise, the *daf-2* mutation induces a significant lifespan extension in the DAF-16::GFP strain, as it does in the wild-type N2 isolate^27^ (Fig. S4b). To validate that the increased DAF-16 nuclear intensity in fact represents transcriptional activity, we analyzed the expression levels of known DAF-16 targets under a subset of DR conditions (Fig. S4d–f) through RT-qPCR. A strong increase in expression was observed for all tested genes, including the robust DAF-16 target, *sod-3*^30^. Additionally, we observed an increase in expression of *mtl-1*, *rpn-6.1*, and *aakg-4*, which are known targets of DAF-16^31^, that also regulate *C. elegans*’ lifespan^32–34^ indicating that DAF-16 migration is activating lifespan regulating genes. This increase in expression of DAF-16 targets was partially inhibited by *daf-16* silencing using RNAi by feeding. Partial inhibition suggests that these genes are also modulated by other transcription factors, or that *daf-16* silencing is inefficient, a likely scenario due to reduced intake of dsRNA-expressing bacteria under DR conditions.

To determine how important the magnitude of DAF-16 activity is in modulating lifespan, we measured mean lifespan under the different DR regimes mentioned previously. All DR regimes lead to an extension in mean lifespan when compared to the control (Fig. 2c–f, Fig. S5) as expected based on prior work^12,35^. In line with the results of DAF-16 activity, the largest lifespan extension was driven by the mid-level concentration of 10^8^ OP50 cells/ml (Fig. 2c, d, Fig. S5), and for 6 and 12 h regimes (Fig. 2e, f, Fig. S5). The 24 h regime also induced lifespan extension, but not as significantly as 6 or 12 h (Fig. 2f). It has been previously observed that long-exposure to starvation increases the likelihood of matricidal hatching, which leads to the animal’s death^36^. While we avoid this potential problem by exposing animals to FUDR, long-exposure to starvation could be inducing deleterious effects that are not fully abrogated by the beneficial pathways activated by DR in *C. elegans*.

### Cumulative lifelong DAF-16 nuclear activity predicts lifespan under DR

**Fig. 3:**
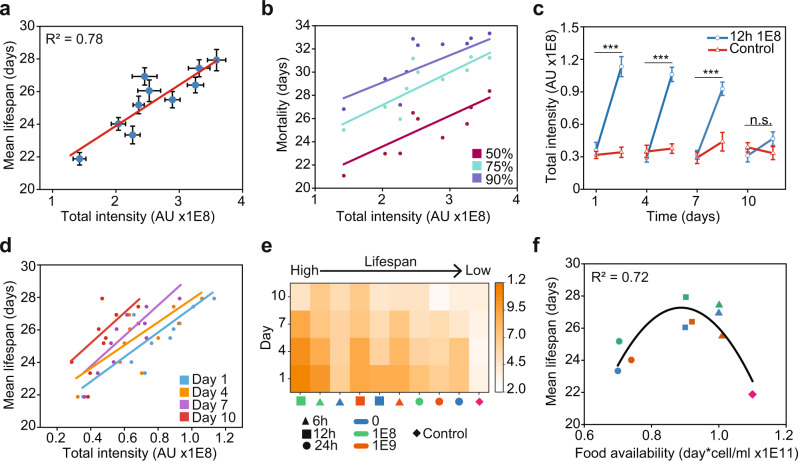
Cumulative lifelong DAF-16 nuclear activity determines lifespan. **a** Mean lifespan of *C. elegans* as a function of lifelong DAF-16 total intensity under various DR regimes. **b** Mortality of *C. elegans* populations as a function of DAF-16 lifelong total intensity. **c** DAF-16 total intensity at a 10^8^ OP50 cells/ml food concentration with 12 h of exposure time compared to an *ad libitum* control at Day 1, 4, 7, and 10 of adulthood. **d** Mean lifespan as a function of DAF-16 total intensity in individual days. **e** Heat map of DAF-16 total nuclear intensity per day under different DR regimes, in order of higher to lower lifespan (left to right). **f** Mean lifespan as a function of total food available during the first 11 days of animal adulthood. 6 h (triangles) and 12 h (squares) regimes are at the peak of the curve while 24 h (circles) regimes and the control (diamond) are at the lower ends of the curve. Error bars are SEM. *p* > 0.05 (n.s.), *p* < 0.001 (***). All *p*-values were calculated using Tukey HSD for all pairwise comparisons after one-way ANOVA (unequal variances) comparison. Linear and quadratic polynomial fits were performed in Origin 2020b.

We assessed the role of DAF-16 cumulative activity in lifespan extension by adding the total intensity of DAF-16 for all identified nuclei per animal at Days 1, 4, 7, and 10 of adulthood, and comparing it to the corresponding mean lifespan (Fig. 3a). We reasoned that the aging process, and in particular longevity, are mostly the outcome of longitudinal exposures during the lifespan^37^. To capture the effects of said exposures we chose the first 10 days of adulthood as the period before a sharp decline in healthspan occurs^38^. Additionally, we reasoned that cumulative total intensity of DAF-16 is the optimal representation of DAF-16 activity during the lifespan as it considers its longitudinal responsiveness. Using linear regression, we found that the cumulative activity of nuclear DAF-16 correlates with mean lifespan with an *R*^2^ of 0.78 for the DR regimes explored in this study. As shown previously, DAF-16 nuclear localization induces expression of aging-regulating genes (Fig. S4), suggesting that lifespan extension due to the DR regimes assessed in this work are mostly modulated by cumulative DAF-16 lifelong spatiotemporal activity. The remainder of lifespan variability could come from DAF-16 activity at days we did not evaluate or, more likely, from the activity of other signaling pathways that also mediate longevity under DR, such as the target-of-rapamycin (TOR) pathway regulated by PHA-4^13,39,40^ and the oxidative stress response regulated by SKN-1^41,42^.

We next evaluated if DAF-16 lifelong activity better correlates with other lifespan metrics. We compared DAF-16 total intensity to mortality quantiles (Fig. 3b) at 50%, 75%, and 90%, which produced *R*^2^ of 0.48, 0.69, and 0.68 respectively. This suggests the DAF-16 response is better captured when a *C. elegans* population perishes once it is already in decline or after 75% of the population has died. Previous studies indicate that DAF-16 has an important role in determining mortality and senescence in *C. elegans*^15^ and *D. Melanogaster*^43^ at their late life stages. Considering this, our results would indicate that cumulative DAF-16 activity is relevant to determine mortality in old animals but not in the case of younger ones.

As mentioned previously, animals underwent DR repeatedly at Days 1, 4, 7, and 10 of adulthood (Fig. 1b). We observed that DAF-16 nuclear activity in animals previously exposed to DR returned to control levels after being well fed for three days. However, DAF-16 responsiveness to DR diminished at each following DR exposure (Fig. 3c). It has been shown that animals that have been previously exposed to DR show increased resistance to stress^44^. DAF-16 responsiveness could possibly be lower on repeated exposure to DR because the animals have already built tolerance to stress from previous exposure. Alternatively, DAF-16 responsiveness could be reduced in aged worms, since the ability of animals to mount a response to stress has been shown to diminish with increasing age^25,45^. By Day 10 of adulthood, once animals have passed their reproductive period, no significant change in DAF-16 activity was observed (Fig. 3c).

We next analyzed the contributions of DAF-16 activity in specific days to mean lifespan (Fig. 3d). Linear regressions of lifespan vs DAF-16 activity from individual days results in an *R*^2^ of 0.71, 0.63, 0.75, and 0.45 at Day 1, 4, 7, and 10 respectively. This analysis indicates that the DAF-16 response relevant for lifespan extension occurs during Day 1, 4, and 7 for the DR regimes analyzed here. Likely, this results from a lack of responsiveness of DAF-16 at Day 10. We analyzed the DAF-16 responsiveness from each day in all DR regimes and sorted the results by their corresponding mean lifespan (Fig. 3e). In all cases, DAF-16 activity is significantly reduced at Day 10, which occurs after animals stopped egg production (at Day 7–8 of adulthood in our experiments). It has been shown that reproduction and lifespan are intertwined in *C. elegans*^46–48^. Potentially, a loss of DAF-16 responsiveness could stem from animals turning-off prioritization of cell protection under stress after their reproductive period is over. To test this idea, we compared the DAF-16 induction levels between animals with and without eggs at Day 8 of adulthood in well-fed and DR animals. DR worms were transferred to an unseeded plate for 6 h. We identified no significant difference in DAF-16 nuclear intensity amongst DR animals with and without eggs (Fig. S6). This data suggests that DAF-16 responsiveness is not linked to egg-laying but other aging factors, or that the loss in DAF-16 responsiveness could be delayed.

We next analyzed if total food availability could also be a good quantitative predictor of mean lifespan. We calculated the total amount of food available to animals up to Day 11 of adulthood (end of the last exposure to DR), in other words we measured the areas under the curve for each DR regime in Fig. S7. Fitting the data with a quadratic polynomial resulted in an *R*^2^ of 0.72 (Fig. 3f), with the highest lifespan values corresponding to intermediate levels of food availability, as expected^3^. This analysis suggests that total food availability, rather than timing or level of food restriction, is more important in modulating lifespan. Presumably, DR regimes different than the ones explored in Fig. 1b, but with similar areas under their curve, could lead to equivalent mean lifespans.

### Intestinal cells and neurons show the largest contribution to DAF-16 nuclear intensity

**Fig. 4:**
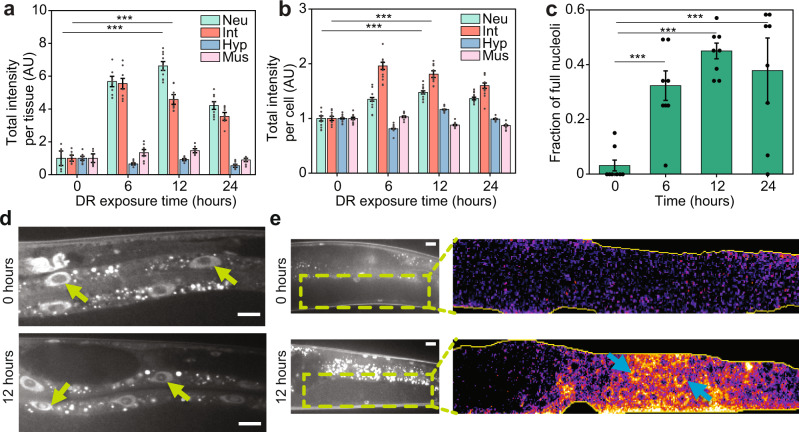
Tissue-specific analysis of DAF-16 reveals crucial role of intestine cells and neurons, nucleolar accumulation, and germline localization under liquid culture DR. **a** DAF-16 normalized total intensity as a function of exposure time for various tissues. Neuron and intestine cells show the largest contribution to DAF-16 total intensity compared to hypodermis and muscle cells at a 10^8^ OP50 cells/ml food concentration. **b** DAF-16 total intensity per cell as a function of exposure time for various tissues at a 10^8^ OP50 cells/ml food concentration. **c** Fraction of intestinal nucleoli showing DAF-16 presence as a function of exposure times at a 10^8^ OP50 cells/ml food concentration. **d** Examples of animals with empty nucleoli (0 h) and DAF-16 in the nucleoli (12 h) at a 10^8^ OP50 cells/ml food concentration. **e** Inset highlights the gonad of a well-fed animal (0 h) and one exposed to a food concentration of 10^8^ cells/ml (12 h). DAF-16 in the germline can be discerned in the DR-exposed animal (light-blue arrows). Error bars are SEM. *p* < 0.001 (***). All *p*-values were calculated using Tukey HSD for all pairwise comparisons after one-way ANOVA (unequal variances) comparison. Scale bars are 20 µM.

Since we observed DAF-16 in a variety of tissues, we then asked which cell-type presents the largest DAF-16 nuclear localization, and better correlates with lifespan extension. Using machine learning, we developed a cell-type classifier to quantify endogenous DAF-16 activity with tissue-specificity. When adding the total intensity of nuclear DAF-16 of all cells of a given type, we observed that neurons and intestinal cells exhibited the largest responses (Fig. 4a). Neurons reach a peak around 12 h, while intestinal cells rapidly peak at 6 h and remain mostly stable afterwards. Since we are quantifying total intensity from all nuclei of each cell type, both the number and size of the nuclei play a role in the comparison of DAF-16 tissue-specific activity. The higher response from neurons could stem from their higher number, with hundreds of neurons per animal as opposed to 20 intestinal cells. To account for this, we analyzed total intensity on a per cell basis. In this case, the largest individual contribution comes from intestinal cells (Fig. 4b), albeit this result could be explained by intestinal cells being the largest cells in *C. elegans*^49^. This is confirmed by neurons having a higher mean intensity than intestinal cells (Fig. S8). From this analysis, we can conclude that the lifespan extension correlation with DAF-16 nuclear localization observed in animals under the DR conditions comes mainly from DAF-16 activity in intestinal cells and neurons. This is further supported by the contributions of total intensity by cell type to mean lifespan (Fig. S9), where we obtained linear regression *R*^2^-values of 0.78, 0.64, 0.47, and 0.00 for neuron, intestinal, muscle, and hypodermal cell types respectively. Note that this low *R*^2^-value for muscle cells is likely the result of an outlier corresponding with the control experiment. These results are in alignment with previous studies indicating that DAF-16 activity in the intestine of *C. elegans* increases the lifespans of *daf-16*(-) insulin/IGF-1-pathway mutants^22^, and that insulin signaling in neurons is important to drive lifespan^50^.

### DAF-16 shows activity in the germline and intestinal nucleoli

During our experimental analysis, we observed that DAF-16 migration was not restricted to cell nuclei but also to the nucleolus in intestinal cells (Fig. 4c, d). We observed that DAF-16 accumulates in 30–50% of intestinal nucleoli under the DR conditions used (Fig. 4c). This phenomenon was not observed with a transgenic strain that expresses DAF-16::GFP under the *daf-16* promoter, MAH97, developed with transgenic approaches that typically result in hundreds of copies of the reporter in a random genomic location. In these conditions, nucleolar localization is likely hidden due to the high brightness of such reporters, suggesting that DAF-16 activity in intestinal nucleoli can only be observed at endogenous levels of expression. The presence of nucleolar DAF-16 in the intestine could be an important player in the mechanisms of lifespan extension observed under DR, as it has been shown that the nucleolus plays an important role in lifespan extension^51–53^. To test this idea, we assessed the correlation between fraction of full nucleoli with mean lifespan, and with DAF-16 total intensity (Fig. S10). We found a correlation (*R*^2^) of 0.49 and 0.58, respectively, suggesting some positive relationship between nucleolar localization and both lifespan and DAF-16 induction. Yet, nucleolar DAF-16 is a weaker predictor than cumulative nuclear abundance. Similarly, we observed DAF-16 in the germline of animals under DR (Fig. 4e), although at extremely dim levels. This presence of DAF-16 was not observed in the MAH97 strain expressing DAF-16 tagged with GFP described above, potentially due to germline silencing of transgenic DNA^54–56^. Tagging the endogenous DAF-16 locus could enable the reporter to bypass these silencing mechanisms.

## Discussion

In this work, we have utilized CRISPR/Cas9 and computer vision to quantitatively analyze the link between the endogenous spatiotemporal activity of the main IIS regulator, DAF-16, and longevity in *C. elegans*. We show that the transcription factor DAF-16 accounts by itself for 78% of the variability in lifespan observed in *C. elegans* under the DR regimes explored here. Previous studies have modulated lifespan by manipulating food concentration or intervening on the genetic pathways governing aging^2,14^. Our quantitative analysis reveals that modulation of a single transcription factor, through DR interventions, accounts for 78% of lifespan variability. Moreover, this work shows that this robust lifespan correlation is achieved by assessing endogenous DAF-16 spatiotemporal activity in a longitudinal manner and is directly affected by the food abundance throughout the animals’ life. Interestingly, we observed that while all DR concentrations induce a response in DAF-16, the maximum DAF-16 response is observed for a mid-level concentration. This non-monotonic response to food concentration has been previously observed^11,57^, and our work suggests this is also true when varying DR exposure time. While prior work has shown that DAF-16 activity does not play a role in starvation-induced longevity when animals are cultured on solid media^58^, our results suggest that starvation does induce DAF-16 activity in liquid media culture. This suggests that DAF-16 could be induced in conditions of increased energy consumption from swimming^26,59^. The regimes that result in the longest mean lifespan show the strongest DAF-16 response (6 and 12 h in Fig. 3e). Notably, the regime of 6 h with 0 OP50 cells/ml exhibits the third longest mean lifespan but its DAF-16 response is low compared to the rest. Brief starvation has been shown to stimulate autophagy and prolong lifespan in *D. Melanogaster*^60^. Potentially, the lifespan extension observed in this particular regime is influenced to a lower extent by DAF-16 while other transcription factors, such as the autophagy modulator PHA-4^13,39^, play a larger role. Additionally, longer DR exposures could result in malnutrition, and lead to reduced DAF-16 responsiveness. Alternatively, DAF-16 could be translocating from nuclei back to the cytoplasm, a phenomenon that has been previously observed during long-term starvation^61^. Notably, the DAF-16 activity that is more relevant for lifespan extension occurred during *C. elegans* reproductive age, highlighting the close relationship between lifespan and reproduction observed in these animals^46^. We explored the activity of DAF-16 in different cell types, showing that intestinal cells and neurons have the largest contributions to DAF-16 activity correlation with lifespan extension. This suggests that the mechanisms that govern DAF-16-dependent longevity in *C. elegans* under DR mainly take place in these cell types. This is emphasized by our finding of DAF-16 migrating into intestinal nucleoli, where some of these lifespan-modulating phenomena could be occurring. While our analysis shows that conditions that induce DAF-16 nuclear localization also induce expression of DAF-16 targets, further examination of a direct causality link between cumulative DAF-16 activity and lifespan extension under DR is warranted.

Finally, our work opens the possibility of further exploration of other genetic pathways, transcription factors, and environmental factors in a holistic manner. For instance, it is unclear if the predictive power of endogenous DAF-16 activity holds under other environmental or genetic perturbations that also modulate lifespan. Moreover, determining whether the contributions to lifespan of other longevity and stress signaling pathways are additive, or if these interact in a synergistic or antagonistic manner will better elucidate longevity modulation in an integrative manner.

## Methods

### Strains, media, and culture

*C. elegans* were maintained on standard Nematode Growth Medium (NGM) plates seeded with OP50 *E. coli* bacteria and kept at 20 °C until they started to lay eggs. Animals were then bleached using standard protocols to obtain age-synchronized populations^62^. This process was repeated three times in total to avoid transgenerational epigenetic effects related to longevity^63^. L4 animals were then transferred to a cell culture flask containing 4 ml of SB media with 10^10^ cells/ml of OP50 bacteria and 100 µM 5-fluorodeoxyuridine (FUDR) and kept at 20 °C for 1 day^64,65^. Cultures were then used for dietary restriction and lifespan experiments. Strains used in this work were ASM10 (del2 [*daf-16*::GFP-C1^3xFlag]) I; ASM33 (del2[*daf-16*::GFP-C1^3xFlag]) I, *daf-2* (e1370) III; and N2 (*C. elegans* wild isolate). OP50 *E. coli* was grown in LB media following standard procedures^66^. Bacteria were washed thrice with SB media, pelletized, and suspended in S-Medium at a concentration of 100 mg/mL, corresponding to a concentration of 2 × 10^10^ cells/ml.

### Generation of transgenic line

We used a CRISPR/Cas9 approach developed by Dickinson et al. to insert a GFP-encoding sequence at the 3' end of *daf-16*^24^. We chose the Cas9 target site by identifying all possible single guide RNAs (sgRNA) in a 200 bp region centered in the stop codon of the target gene. This region was selected to permit introduction of the fluorescent tag at the C-terminus, tagging in this manner all possible isoforms of the gene. Criteria such as specificity, activity, and distance to the stop codon were considered in selecting the sgRNA using the GuideScan design tool^67^. This 20 bp sequence was introduced into a Cas9-encoding construct (pDD162, Addgene #47549) using a NEB Q5 Site-Directed Mutagenesis Kit to generate plasmid pJHR1. 500-700 bp long homology arms at each side of the *daf-16* stop codon were then generated by PCR amplification of genomic DNA from N2 animals. These fragments were isolated, purified, and introduced in a construct containing a selection cassette (pDD282, Addgene #66823) using NEBuilder HiFi DNA Assembly Mix to generate plasmid pJHR2 (Table S1, Fig. S1, S2). A mix containing 15 ng/μL pJHR1, 50 ng/μL pJHR2, and 2.5 ng/μL pCFJ90 (mCherry co-injection marker) was injected in the gonads of 100 N2 young-adult animals following standard microinjection procedures^68^. These animals were transferred to NGM plates and left to produce progeny at 25 °C. Hygromycin was added to the plates to kill untransformed F1 progeny. After 7 days, animals showing the Rol phenotype and lacking red fluorescent extrachromosomal array markers were transferred to new NGM plates without hygromycin. Individual putative knock-in animals were transferred to new plates for homozygous selection. Finally, L1 larvae from homozygous plates (those that contained only animals with the Rol phenotype) were heat-shocked at 34 °C for 4 h to induce Cre expression, and excision of the selection cassette. Wild-type adult animals lacking the Rol phenotype were picked and maintained as the strain containing the fluorescent-tagged gene. PCR and Sanger sequencing were performed to confirm correct introduction of the tag.

### Dietary restriction and fluorescence imaging

Approximately 200 Day 1 adult worms in liquid culture were washed three times with SB media and moved to a flask containing 4 ml of SB media with a reduced food concentration to induce DR (10^9^, 10^8^ or 0 OP50 cells/ml) for a fixed amount of time (6, 12, or 24 h). After the DR period concluded, additional OP50 was added to raise the concentration to an *ad libitum* level (10^10^ OP50 cells/ml). FUDR concentration was raised to 100 µM total concentration to prevent offspring production^69^. Worms were then kept at 20 °C for 3 days at *ad libitum* food concentration. DR exposure was repeated on Days 4, 7, and 10 of adulthood. At the start and end of each DR exposure, ~20–30 worms were immobilized using 10 µL of 2 mM tetramisole on dried 2% agarose pads of ~1 cm diameter. Confocal fluorescence microscopy was performed with a Leica DMi8 microscope paired with an 89 North LDI Laser Diode Illuminator coupled with a CrestOptics X-Light V2 confocal imager and an Orca-Flash 4.0 digital CMOS camera. The exposure time and laser intensity were kept constant throughout all experiments at 100 ms and 100%, respectively. Images containing 30 slices (Z-stacks with 3 µm spacing) were acquired with a MATLAB GUI (Graphical User Interface). Images were acquired in the green and red channels sequentially. This enabled later subtraction of autofluorescence present in both channels with a custom MATLAB script.

### DAF-16 localization in liquid vs solid culture

Age-synchronized animals were grown from embryo to L4 larvae on NGM plates containing OP50 bacteria. At this stage, half of the animals were washed from plates to a 15 ml conical tube and left to settle to the bottom. After three cycles of washing and discarding the supernatant, animals were moved to flasks containing 4 ml of SB media with a concentration of 10^10^ OP50 cells/ml. Flask were put on a shaker at 20 °C for 3 h, after which both the animals still on plates and those in liquid culture were imaged as described in the previous section.

### Lifespan experiments

Approximately 200 worms per flask were kept in liquid culture in the same conditions described in the previous section. Animal mobility was analyzed in a stereoscope through visual inspection. Nematodes were scored as alive (active and swimming), dead (rigid and not moving), and censored (lost to manipulation). This was performed every 3rd day, starting on Day 1 of adulthood. Lifespan curves were constructed using Online Application for Survival Analysis 2^70^ to obtain the mean lifespan for each population. For lifespan in the *daf-2* (ASM33) vs wildtype (ASM10), we performed this analysis on solid NGM plates with 150 µM FUDR (5-Fluoro-2'-deoxyuridine) and seeded with OP50 until Day 10. Following Day 10, worms were placed on solid NGM plates without FUDR. Animals were transferred to avoid depletion of the OP50 bacterial lawn. Nematodes were scored as alive if movement was observed upon gentle touching with a worm pick.

### Quantitative image processing

Images were analyzed using a MATLAB script, customized to segment cell nuclei in each slice of the z-stack. The algorithm’s most important steps are: (1) Subtraction of red channel image from green channel image to eliminate autofluorescence present in both images; (2) Generation of a binary mask from the subtracted image; (3) Identification of nuclei by retaining segmented objects that: (a) filled up at least 50% of their bounding box, (b) had eccentricity values under 0.85, and (c) had solidity values above 0.75. The threshold values were determined based on visual assessment of the characteristics of nuclei. Object characteristics were extracted using the regionprops function; (4) Use of the imopen function to smooth edges. (5) Deletion of objects smaller than neurons and larger than intestinal cells using the bwareaopen function. These operations enable for the selection of nuclei (ellipse or circle-like objects) while eliminating other features such as autofluorescence, animal edges, embryos, etc. in a stepwise manner (Fig. 1a). To avoid over-estimation, nuclei present in multiple slices were identified by comparing their centroids. If multiple centroids with similar values in the ±4 pixels range were identified in multiple slices, only the object with the largest area was retained for properties extraction. The MATLAB regionprops function was then used to quantify the total intensity in the segmented nuclei and the data extracted was stored in a MATLAB nested structure. Total intensity was calculated as the sum of the intensity of the pixels from all cell nuclei extracted per worm.

### Cell-type classification and nucleolus analysis

To classify nuclei belonging to specific tissues, we used a Fine Tree multi-class classification algorithm. A ground truth set of 600 cell nuclei and their corresponding class (intestine, neuron, hypodermis, or muscle) was manually generated. The Fine Tree algorithm was trained using MATLAB Classification Learner, by using the following object properties as features: area, eccentricity, and equivalent diameter. The generated Fine Tree algorithm was tested against a validation image set of 200 cell nuclei. This resulted in a 96.1% accuracy of the Fine Tree algorithm compared to the manually assessed nuclei. Both the ground truth and validation sets contained nuclei from animals at all the ages evaluated in this work. For nucleolar analysis, DAF-16-filled nucleoli in intestinal cells were counted manually using FIJI. A nucleolus was considered empty when the nucleus’ center was empty. A nucleolus was considered filled when the nucleus’ center had fluorescence due to GFP-tagged DAF-16.

### RNAi by feeding

Age-synchronized worms were grown from the egg stage to the L4 stage at 20 °C on NGM plates containing HT115 bacteria. Animals were then transferred to flasks containing HT115 bacteria with an empty vector (control) or HT115 bacteria carrying the *daf-16* RNAi vector from the Ahringer library (acquired from Source Biosciences)^71^, and grown until Day 1 of adulthood. Animals were then exposed to DR for 6 or 12 h at a food concentration of 10^8^ HT115 cells/ml (control or *daf-16* RNAi).

### RNA isolation and quantitative PCR (qPCR)

For RNAi experiment: approximately 50 animals that were previously subjected to RNAi treatment (control or *daf-16* RNAi) were transferred to Trizol (Invitrogen) and vortexed twice for 30 s. Total RNA was isolated using Direct-Zol RNA MicroPrep Kit (Zymo Research) according to the manufacturer’s protocol. Quantitative PCR was performed on a CFX384 Touch Real-Time PCR Detection System (Bio-Rad Laboratories) using Luna Universal One-Step RT-qPCR Kit (New England BioLabs) according to the manufacturer’s protocol. RT-qPCR data was analyzed using the ∆∆Ct method. Target genes were selected using the FOXODB database of DAF-16 direct targets^31^. Gene expression levels were normalized using *ama-1* as housekeeping gene. For *daf-2* experiment: approximately 300 animals subjected to *ad libitum* conditions (ASM10 and ASM33) were washed to minimize OP50 contamination, transferred to Trizol (Invitrogen) and homogenized using a motorized pestle twice for 30 s, and incubated for 30 s prior to purification. Total RNA was isolated using Direct-Zol RNA MiniPrep Plus Kit (Zymo Research) according to the manufacturer’s protocol, extending DNase I treatment to 45 min. Additional purification using a TURBO DNA-*free* kit (Invitrogen) was performed on the isolated RNA according to the manufacturer’s protocol. Two-step quantitative PCR was performed on a CFX384 Touch Real-Time PCR Detection System (Bio-Rad Laboratories) using a LunaScript RT SuperMix kit (New England BioLabs) and the Luna Universal qPCR Master Mix kit according to the manufacturer’s protocol. The qPCR data was analyzed using the ∆∆Ct method. Gene expression levels were normalized using *act-1* as a housekeeping gene.

### Analysis of egg-laying and DAF-16 induction

Well-fed worms were cultured on solid NGM plates for three generations. For analysis, worms were age synchronized and cultured on NGM plates with OP50 until Day 8 of adulthood. Animals were transferred to separate adults from progeny. For dietary restricted animals, worms were transferred to an unseeded plate for 6 h. Animals were then imaged as detailed above, while also including a bright-field z-stack to analyze egg content. Animals with eggs clearly visible were classified as positive for eggs.

### Statistics and reproducibility

Linear regressions were performed in Origin 2020b using the FitLinear function. Polynomial fits were performed in Origin 2020b using the FitPolynomial function. Significance tests such as t-Test and ANOVA were performed using the Data Analysis Add-on in MS Excel 2016. Lifespan analysis was performed with the Online Application for Survival Analysis 2^70^ by using number of dead and censored animals as input; obtaining mean lifespan with a Kaplan–Meier estimator.

### Reporting summary

Further information on research design is available in the Nature Portfolio Reporting Summary linked to this article.

## Supplementary information

Supplementary Information FINAL

Reporting Summary

## Data Availability

All data and the code used in this manuscript has been deposited in Dryad (10.5061/dryad.vx0k6djwt).
