# Supplementary material for: Endogenous DAF-16 spatiotemporal activity quantitatively predicts lifespan extension induced by dietary restriction
Source: Commun Biol. 2023 Feb 20;6:203. doi: 10.1038/s42003-023-04562-2 (PMC9941123; doi:10.1038/s42003-023-04562-2)
Supplement: Supplementary file 1 — Supplementary Information FINAL [file 42003_2023_4562_MOESM1_ESM.pdf]

Endogenous DAF-16 Spatiotemporal Activity Quantitatively Predicts Lifespan Extension Induced by Dietary Restriction

Javier Huayta[1], Joseph P. Crapster, and Adriana San-Miguel[1,*]

SUPPLEMENTARY FIGURES AND TABLES

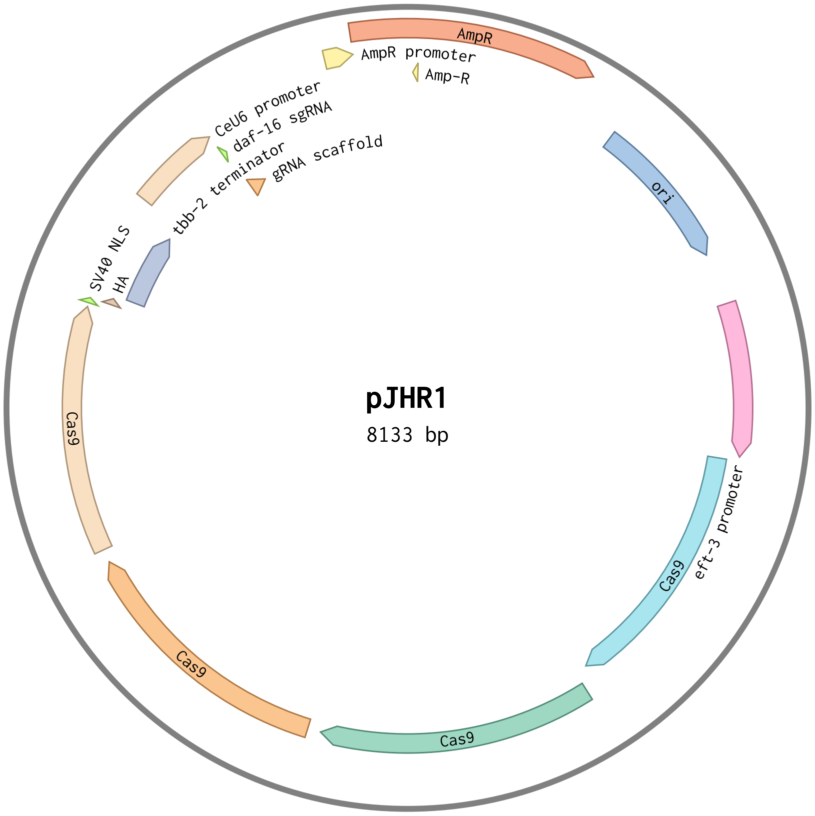

Figure S1: Plasmid used for generation of transgenic strain with CRISP/Cas9. pJHR1 contains the sgRNA targeting DAF-16 endogenous locus.

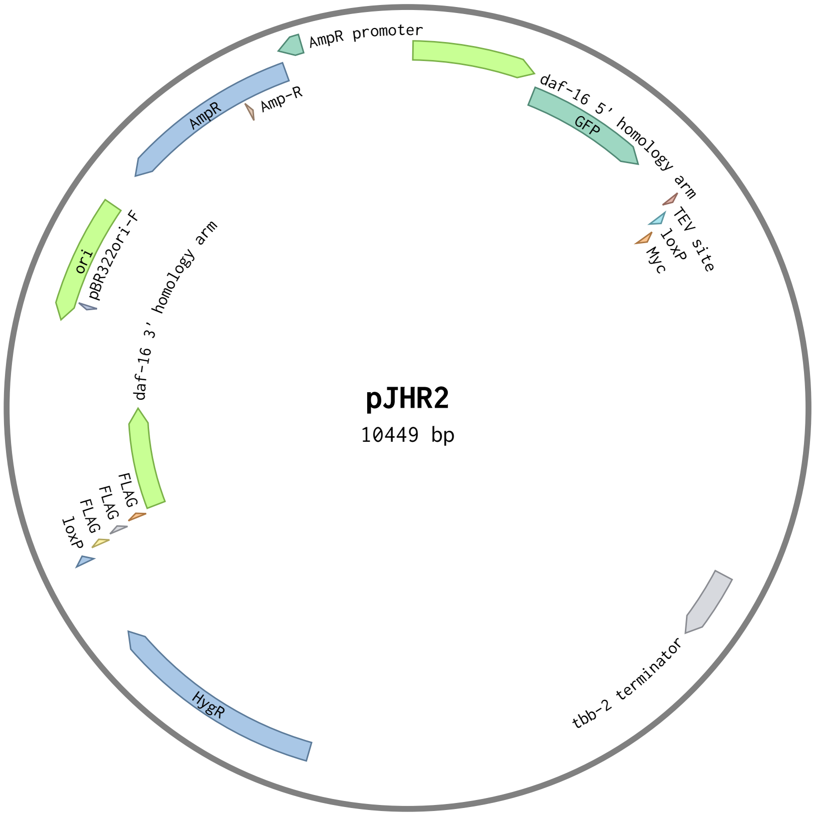

Figure S2: Plasmid used for generation of transgenic strain with CRISPR/Cas9. pJHR2 contains GFP, self-excising cassette, and homology arms for DAF-16 tagging.

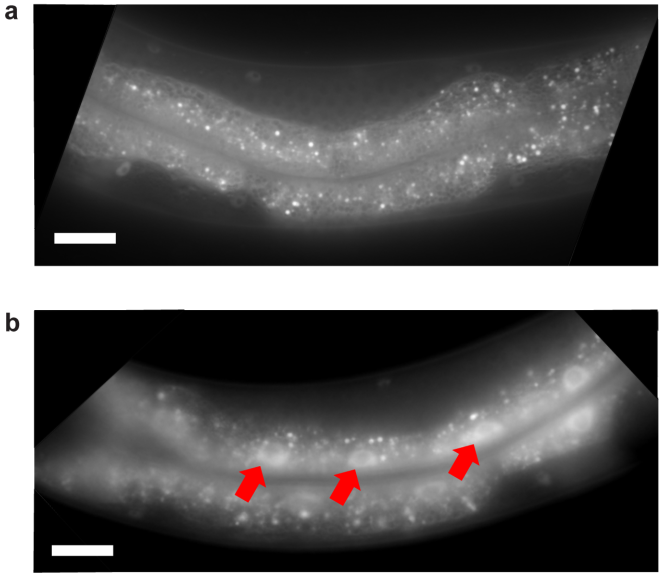

Figure S3: DAF-16 localizes to nuclei in liquid culture. A) Intestine of a young adult animal grown on solid media. B) DAF-16 localized in intestinal cell nuclei of young adult animals 3 hours after being transferred from solid media to liquid culture (red arrows). Scale bars are 20 μM.

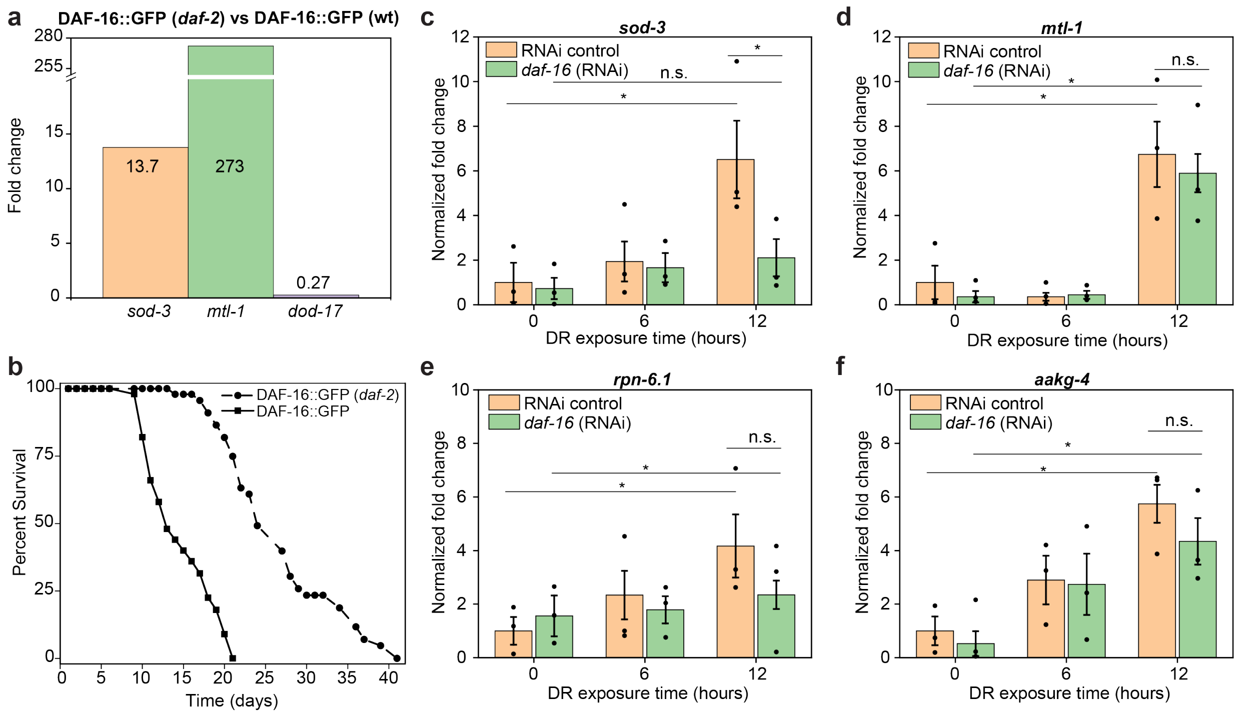

Figure S4: DAF-16::GFP is functional and DAF-16 target genes increase under DR. A) Constitutive activation of DAF-16::GFP through a *daf-2 (e1370)* mutation induces *sod-3* and *mtl-1*, and inhibits *dod-17*. B) DAF-16::GFP with a *daf-2 (e1370)* mutation live longer. C-F) Normalized fold change for *sod-3*, *mtl-1*, *rpn-6.1*, and *aakg-4* under a food concentration of 10^8 HT115 cells/ml for 6 and 12 hours. Error bars are SEM. $p > 0.05$ (n.s.), $p < 0.05$ (*). All p-values were calculated using Tukey HSD for all pairwise comparisons after one-way ANOVA (unequal variances) comparison.

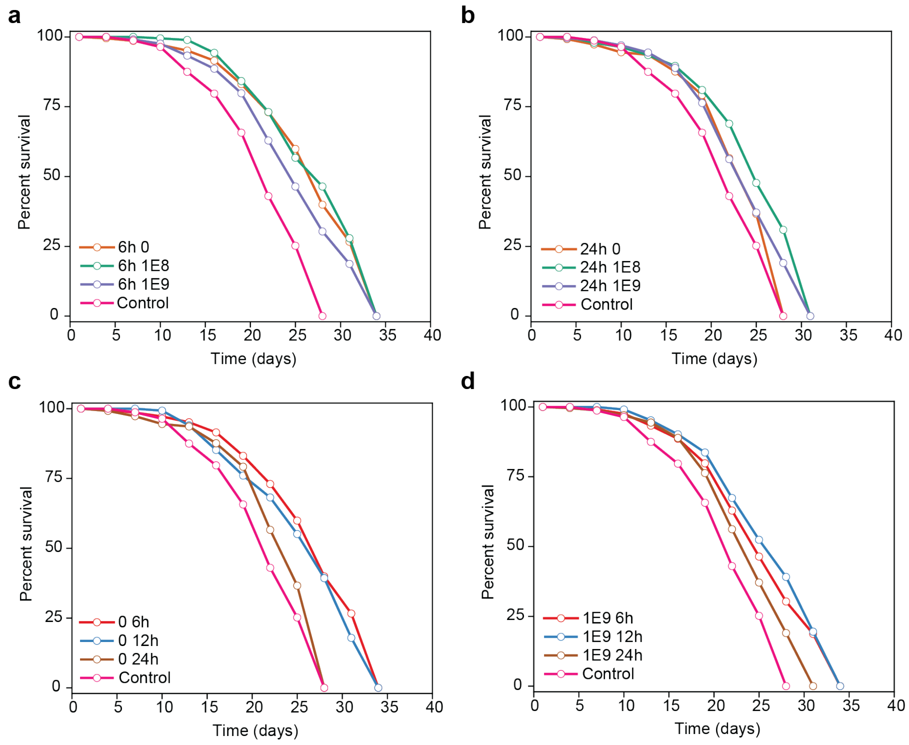

Figure S5: Lifespan curves for various DR regimes. A) Varying food concentration with 6 hours exposure time. B) Varying food concentration with 24 hours exposure time. C) Varying exposure time with a food concentration of 0 OP50 cells/ml. D) Varying exposure time with a food concentration of 10^9 OP50 cells/ml.

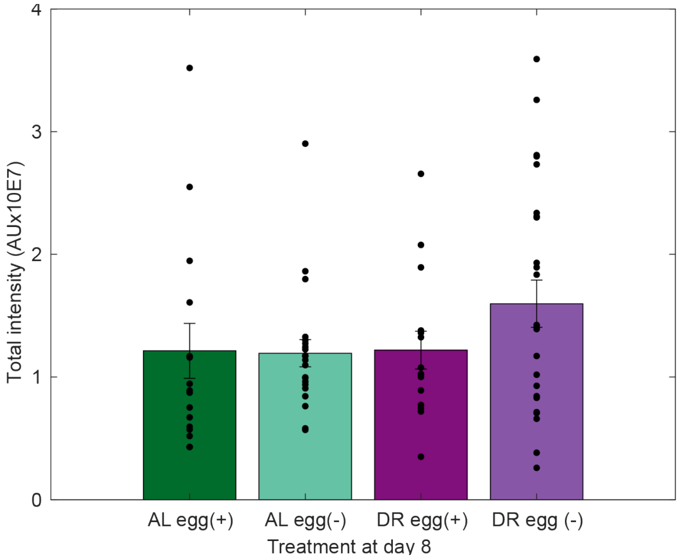

Figure S6: DR induces DAF-16 nuclear localization before egg laying stops. Adult animals (Day 8) exposed to *ad libitum* (AL) food show no difference in DAF-16 nuclear localization regardless of presence of eggs. Adult animals (Day 8) with eggs under 6 hour DR (no food) show increased DAF-16 nuclear localization compared to animals without embryos under the same DR conditions. Error bars are SEM. $p < 0.05$ (*). All p-values were calculated using Tukey HSD for all pairwise comparisons after one-way ANOVA (unequal variances) comparison.

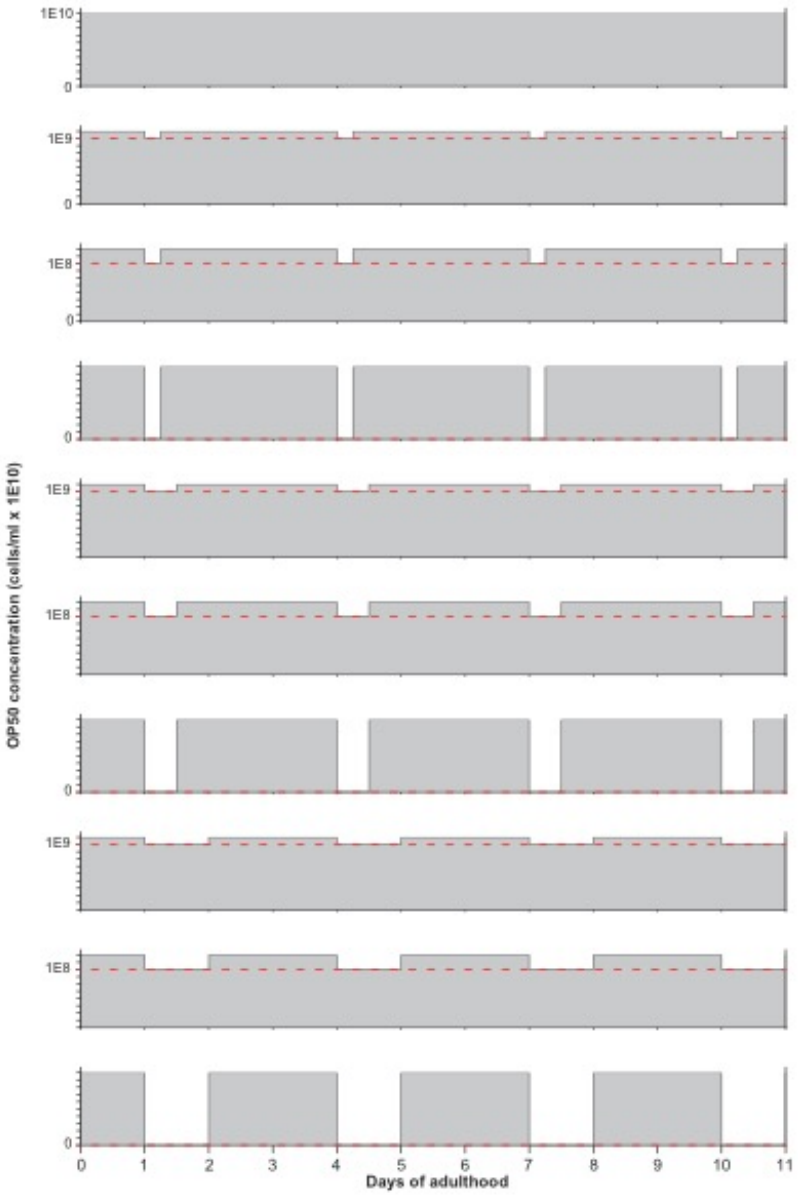

Figure S7: Graphic representation of food availability for each of the DR regimes evaluated. Grey areas were calculated for each DR regime and used in Figure 3F.

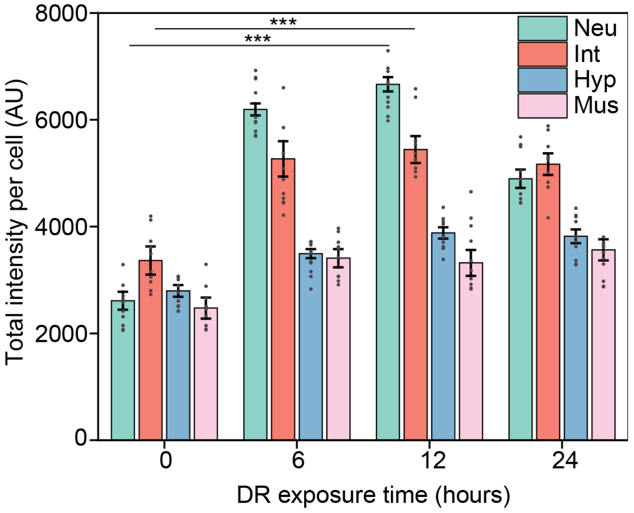

Figure S8: Mean intensity per cell type at various exposure times. Intestinal cells and neurons show the largest mean intensity compared to other cell types. Error bars are SEM. $p < 0.001$ (***). All p-values were calculated using Tukey HSD for all pairwise comparisons after one-way ANOVA (unequal variances) comparison.

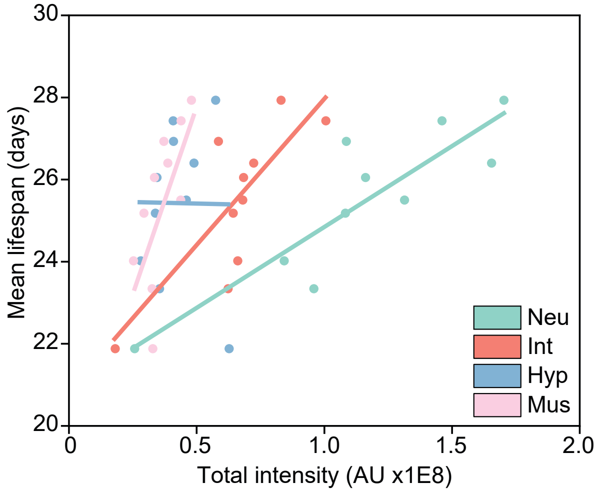

Figure S9: Mean lifespan dependence on total intensity by cell type. Linear regression analysis of mean lifespan under DR as a function of DAF-16 total nuclear intensity in specific cell types indicates that the best correlation corresponds to neurons ($R^2 = 0.78$) and intestinal cells ($R^2 = 0.64$) with minor or negligible contributions from hypodermal ($R^2 = 0.47$) and muscle cells ($R^2 = 0.00$). Linear fits were performed in Origin 2020b.

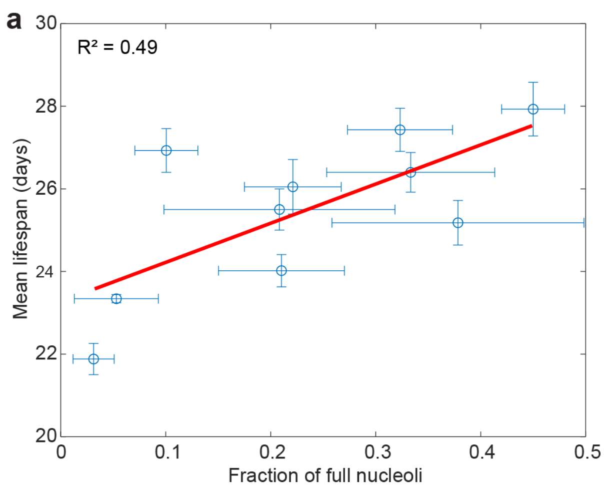

Figure S10: Mean lifespan and DAF-16 total intensity as a function of full nucleoli. A) Linear regression analysis of mean lifespan as a function of full intestinal nucleoli shows a trend with $R^2 = 0.49$. B) Linear regression analysis of DAF-16 total intensity as a function of full intestinal nucleoli shows a directly proportional trend with $R^2 = 0.58$. Linear fits were performed in Origin 2020b.

Table S1: Primers used for generation of transgenic line ASM10 *daf-16* (del2 [*daf-16*::GFP-C1^3xFlag])

I Description	Primer
3' forward homology arm in pDD282	CGTGATTACAAGGATGACGATGACAAGAGA TAAATTCTCTTCATTTTGTTTCCCC
3' reverse homology arm in pDD282	GGAAACAGCTATGACCATGTTATCGATTTCG GCTGTGATGATCGTTGAGTG
5' forward homology arm in pDD282	ACGTTGTAAAACGACGGCCAGTCGCCGGCA TACGGGCTCGATTTTCGTGA
5' reverse homology arm in pDD282	CATCGATGCTCCTGAGGCTCCCGATGCTCCC AAATCAAAATGAATATGCTGtCCT
sgRNA added to pDD162	ATGAGCTGAGTCAAGCTGGA
Forward to introduce sgRNA in pDD162	ATGAGCTGAGTCAAGCTGGAGTTTTAGAGC TAGAAATAGCAAGT
Reverse to introduce sgRNA in pDD162	CAAGACATCTCGCAATAGG
